# E-scouts in support of online teaching during the SARS-CoV-2 pandemic

**DOI:** 10.3205/zma001367

**Published:** 2020-12-03

**Authors:** Chantal Klemmt, Sarah König

**Affiliations:** 1 Universitätsklinikum Würzburg, Institut für Medizinische Lehre und Ausbildungsforschung, Würzburg, Germany

**Keywords:** e-scouts, digitalization, teaching during the SARS-CoV-2 pandemic, online teaching

## Abstract

**Introduction: **During the SARS-CoV-2 pandemic, students with an affinity to digital technology supported the online teaching activities of lecturers on an ad-hoc and needs-oriented basis. The aim of the study was to determine the necessity and acceptance of these so-called e-scouts.

**Methodology: **An online survey was sent via the Faculty of Medicine mailing list of department heads and teaching coordinators. Thirty valid responses were identified and evaluated.

**Results: **The e-scouts provided support in particular with preparing audio commentaries of presentation slides, video recording of presentations, and the implementation of case-based e-learning. The main reasons for requesting help from the students were technical support, equipment loan, and support with how to use the learning platform and course management system. Overall, the lecturers rated the service provided by e-scouts as highly satisfactory and praised their prompt and competent assistance. Over 50% of the lecturers stated their wish to continue working with e-scouts in the future and integrate online activities into their teaching even beyond the SARS-CoV2 pandemic.

**Discussion and conclusion: **With the support of the e-scouts, lecturers received assistance with developing and improving their own media competence without bureaucratic hurdles, aiming to create online courses for the local teaching and learning platform. Thereby, the e-scouts’ perspective was very helpful in providing both important insights into how students learn as well as impulse toward the further development of digital teaching activities.

## Zusammenfassung

**Einleitung: **Technikaffine Studierende unterstützten adhoc und bedarfsorientiert die Online-Lehraktivitäten der Dozierenden während der SARS-CoV-2-Pandemie. Ziel der Studie war es, die Nutzung und Akzeptanz der sogenannten E-Scouts zu eruieren.

**Methodik: **Eine Online-Umfrage wurde über den Verteiler der Leitungspositionen und Lehrverantwortlichen gesendet, es konnten 30 gültige Bögen identifiziert und deskriptiv ausgewertet werden.

**Ergebnisse: **Die E-Scouts unterstützten insbesondere bei der Vertonung von Präsentationsfolien, der Aufzeichnung von Präsentationen und bei E-Learning-Fällen. Gründe für die Inanspruchnahme stellten vor allem die technische Unterstützung, die Ausleihe von Equipment und die Unterstützung mit Lernplattformen und Kursmanagementsystemen dar. Insgesamt beurteilten die Dozierenden die E-Scouts mit einer hohen Zufriedenheit und lobten über eine zeitnahe und kompetente Hilfestellung. Über 50% der Dozierenden möchte auch in Zukunft auf E-Scouts zugreifen und Online-Aktivitäten über den Zeitraum der SARS-CoV2-Pandemie in die Lehre integrieren.

**Diskussion und Schlussfolgerung: **Durch die Unterstützung der E-Scouts konnten Dozierende rasch und ohne bürokratische Hürden zu einer Medienkompetenz verholfen werden, um verschiedene Online-Lehrangebote über die Lehr- und Lernplattform der Universität zur Verfügung zu stellen. Die Perspektive der Studierenden hat dabei sehr geholfen, die Orientierung an den Lernenden herbeizuführen und wichtige Impulse für die weitere Entwicklung von digitalen Lehraktivitäten zu geben.

## 1. Introduction and objectives

With the announcement that the summer semester of 2020 would be online, the Faculty of Medicine in Würzburg established the concept of e-scouts to enable online teaching and deal with the restrictions caused by the SARS-CoV-2 pandemic. The intention was to the implement online teaching activities by minimizing the technical efforts and to balance out the heterogeneous digital competence of teachers [1]. The e-scouts comprised five students of the human medicine and dentistry degree courses who had a special interest in digitalization of teaching and creating suitable learning materials. Initially, they were trained in how to use the local learning platform and how to employ technical principles and didactic strategies of online teaching. The task of the e-scouts was to provide teaching staff with ad hoc and needs-oriented assistance in planning and designing online activities for the semester. Lecturers were able to contact the e-scouts by e-mail or telephone. After being provided with the necessary equipment, they visited the lecturers in their working and teaching environment to support them with basic IT knowledge and more sophisticated technical projects. The e-scouts were funded by the Office of the Dean of Studies, so there were no administrative or financial restrictions.

The aim of this study was to evaluate the necessity and acceptance of e-scouts as experienced by teaching staff. In particular, the satisfaction with their work, as well as the frequency and reasons for consultation were determined. Furthermore, the need for further help beyond the end of the online semester was assessed.

## 2. Method

The questionnaire comprised 18 items: addressing the demographics of the lecturers and the curricular implementation of their courses, the support provided by the e-scouts, and further intention to use and develop online activities by the teaching staff. The online survey was run on the evaluation platform EvaSys (EvaSys®, Lüneburg, Germany); department and institute heads of the Faculty of Medicine as well as teaching coordinators were invited to participate (N=96). Furthermore, the primary participants were asked to forward the survey to other lecturers. Open text answers were evaluated and categorized thematically. Quantitative data were analyzed using descriptive statistics. A five-level Likert scale was employed to rate items (1=don't agree at all to 5=fully agree); mean values (M) and standard deviation (SD) were calculated. 

## 3. Results

A total of 30 valid responses were analyzed; differing numbers are given in brackets. 55% of the respondents were female (n=29). On average, lecturers had 16 years of teaching experience in higher education (n=27). 90% of the respondents were mainly engaged in the degree course of human medicine and 70% were predominantly involved in clinical teaching (n=27).

The e-scouts were positively evaluated for their timely responses to enquiries (n=22; M=4.5; SD=0.7) and their ever helpful digital competence (n=22; M=4.4; SD=0.8). In terms of frequency (n=29), 34.5% of respondents stated that they worked with e-scouts regularly during the semester, 37.7% only at the beginning of the semester and 27.6% never. The three most frequent support reasons were assessed: production of audio commentaries for presentation slides (53.3%), video-recording of presentations (23.3%) and the implementation of case-based e-learning (23.3%). The three most important methodological reasons for using the service were technical support (e.g. web-conferencing by Zoom or Skype) (53.3%), loan of equipment (e.g. headsets) (16.7%) and support with the learning platform and course management system (e.g. WueCampus) (16.7%). 

**Figure 1:**
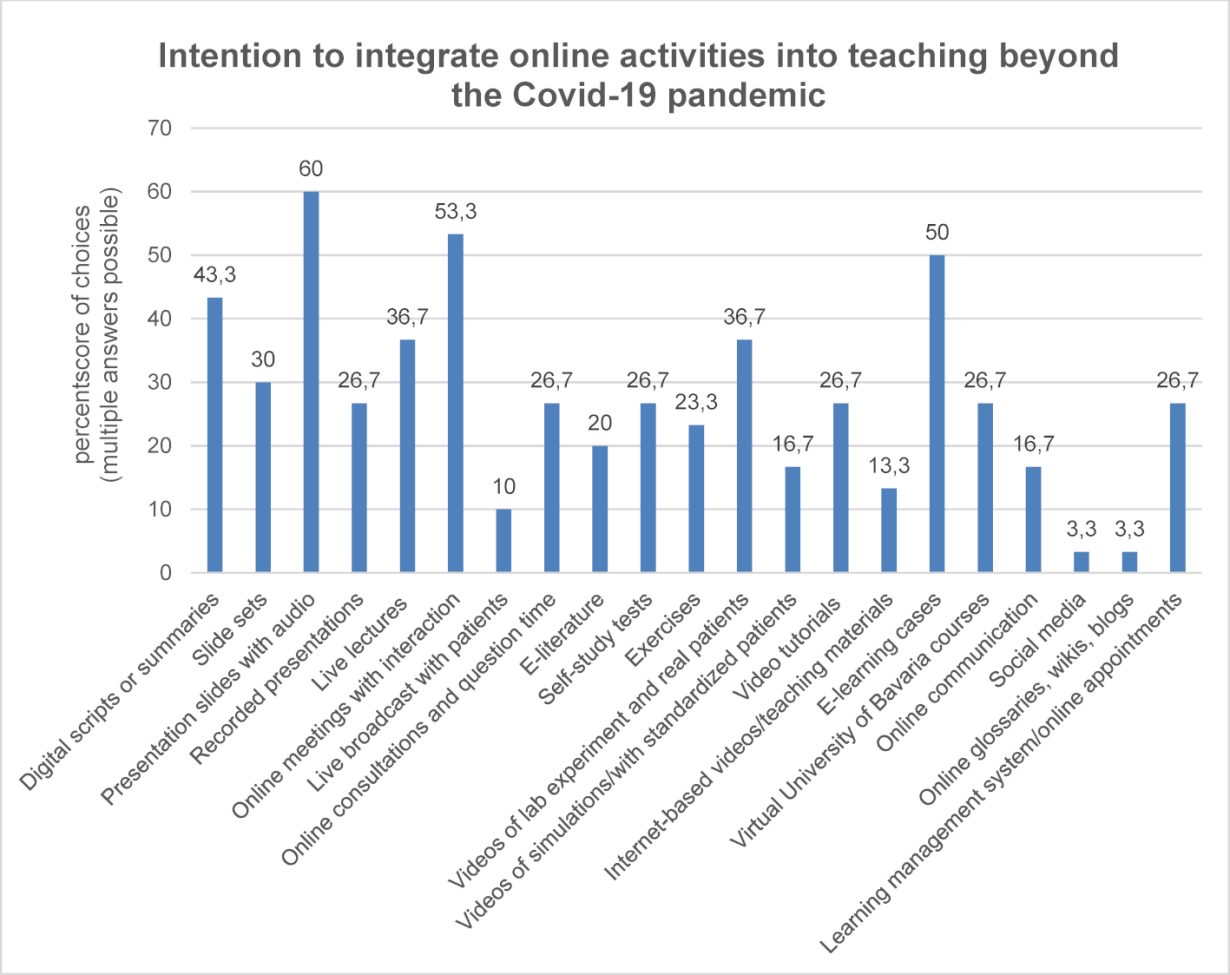
Overview of the online activities (teaching and learning formats/learning materials) that are intended to be integrated into the teaching of the respondents in the future beyond the Covid-19 pandemic. The relative proportion of the total number (N=30) of answers is plotted on the Y-axis.

The majority of teaching staff stated that they would request further support beyond the summer semester (n=26; mw=4.5; s=0.8). The possible option to replace the traditional teaching formats in the acquisition of knowledge (e.g. lectures, seminars) with online teaching was rejected by 17.2% of respondents, whereas 24.1% (n=29; M=3.2; SD=1.4) fully supported the idea. Figure 1 (Fig. 1) illustrates online activities that were recommended for prospective integration into medical education beyond the SARS-CoV-2 pandemic. In particular, presentations with embedded audio, online teaching with interaction and e-learning cases were emphasized. 55.5% of the respondents were capable of independently conducting online teaching (n=18; M=3.6; SD=0.9). 

The clustering of the open answer questions pointed out what the lecturers appreciated the most about the concept of e-scouts: prompt and rapid support, which often focused on the pragmatic and successful technical implementation of digital teaching material, image processing included. E-scouts were commended for being easily contacted, competent and committed to the cause. The turnover of contact persons and supplementary advice on technical as well as didactic concepts were noted as areas with room for improvement.

## 4. Conclusion and outlook

All in all, e-scouts are viewed as highly accepted. Among other things, conceptual advice on the opportunities of online teaching was requested as optimization. Since e-scouts provided essential technical support in over 50% of cases this semester, the conceptual consulting aspect will be focused in the coming semester, in order to adapt the didactic design to the requirements of online formats [2]. The regular use of e-scouts by 35% of the respondents and 38% at the beginning of the semester was pleasing. In addition, the majority expressed their desire for further support from e-scouts. Only slightly more than half of the respondents felt that they would be able to continue online teaching independently. We take this as an opportunity to continue with the service e-scouts provide the teaching staff. In advance of the further planning of any future hybrid semester under the conditions of a pandemic phase of low incidence (online teaching supplemented by selected face-to-face teaching), a survey of the demand has already been started. 

It should be emphasized that e-scouts were an important personnel tool to remove some of the technical hurdles that still existed in some cases and to raise the level of digital teaching skills of the teaching staff. The e-scouts were often the decisive initial impulse for teachers to explore the possibilities of online teaching [3], [4]. As a result, teaching staff have continued to develop further digital teaching activities independently. The use of e-scouts can therefore be seen as empowerment of the teachers.

Although the number of participants allows only limited conclusions to be drawn, the evaluation study provides important information on further support by e-scouts and the need for development to integrate online activities into teaching sustainably.

## Competing interests

The authors declare that they have no competing interests.
